# Supplementary material for: CLEMSite, a software for automated phenotypic screens using light microscopy and FIB-SEM
Source: J Cell Biol. 2022 Dec 23;222(3):e202209127. doi: 10.1083/jcb.202209127 (PMC9802685; doi:10.1083/jcb.202209127)
Supplement: Table S1 — shows RMSE of targeting position. [file JCB_202209127_TableS1.docx]

Table 1: RMSE of targeting position



1 2 3

RMSE Global RMS Local (estimated) RMS Local (measured)

| S1 Spots (n=53) | 6.44 *±* 4.3 | 4.53 *±* 3.4 | 4 *±* 1.9 |
| --- | --- | --- | --- |
| S2 Spots (n=46) | 9.62 *±* 5.1 | 4.26 *±* 3.1 | 4.94 *±* 3.5 |
| S1 COPB1 (n=33) | 18.76 *±* 11.5 | 12.73 *±* 10 | 12 *±* 4.3 |
| S2 COPB1 (n=47) | 20.56 *±* 13.5 | 14.98 *±* 13.5 | 9.86 *±* 6.5 |
| Avg and std (n=179)* | 13.21 *±*6.2 | 8.71*±* 5.2 | 7.7*±*4.4 |

* except for the last column, in which n = 40

(1) RMSE in *µm* of global transformation using all samples in an affine transform, (2) RMSE in *µm* using a transformation involving only local samples (8 closest landmarks), (3) RMSE in *µm* measured by manual registration of images. Only *n* = 10 of the images were used per experiment

1
