# Supplementary material for: CLEMSite, a software for automated phenotypic screens using light microscopy and FIB-SEM
Source: J Cell Biol. 2022 Dec 23;222(3):e202209127. doi: 10.1083/jcb.202209127 (PMC9802685; doi:10.1083/jcb.202209127)
Supplement: Table S2 — shows description of the 32 siRNA spots added to one dish in Case Study 1, referring to Fig. 4. [file JCB_202209127_TableS2.docx]

1

**Supplementary Table 2: Description of the 32 siRNA spots added to one dish in Case Study 1, referring to Fig. 4.**

|  | Gene name | Full name | Gene description | siRNA ID  (silencer select  Ambion) | Sense siRNA Sequence | Antisense siRNA Sequence | Golgi phenotype |
| --- | --- | --- | --- | --- | --- | --- | --- |
| 1* | COPB2 | coatomer protein  complex, subunit beta 2 | Coatomer complex required for budding from Golgi membranes and essential for the retrograde Golgi-to-ER transport of dilysine tagged proteins | s17738 | CGAUGUAUCUCCUAGGCUAtt | UAGCCUAGGAGAUACAUCGtc | Diffuse |
| 2 | WDR75 | WD repeat domain 75 | Ribosome biogenesis factor | s38530 | CAGCUAGCAAAGAUGGUUAtt | UAACCAUCUUUGCUAGCUGta | Condensed^✝^ |
| 3* | DNM1 | dynamin 1 | Dynamin subfamily of GTPbinding proteins, involved in clathrin-mediated endocytosis and other vesicular trafficking processes | s144 | GCAGUUCGCCGUAGACUUUtt | AAAGUCUACGGCGAACUGCtg | Fragmented |
| 4* | COPG1 | coatomer protein  complex, subunit gamma | Coatomer complex required for budding from Golgi membranes and essential for the retrograde Golgi-to-ER transport of dilysine tagged proteins | s22431 | CGUCGGAUGUGCUACUUGAtt | UCAAGUAGCACAUCCGACGga | Diffuse |
| 5 | C1S | complement  component 1, s subcomponent | Encodes a serine protease, which is a major constituent of the human complement subcomponent C1. | s2157 | CCAAGUCCCAUACAACAAAtt | UUUGUUGUAUGGGACUUGGaa | Tubular^✝^ |
| 6 | DENND4C | DENN/MADD domain containing 4C | Guanine nucleotide exchange factor (GEF) activating RAB10. Promotes the exchange of GDP to GTP. | s31214 | GUUUGGACCUUCCUAGUAAtt | UUACUAGGAAGGUCCAAACgt | Fragmented^✝^ |
| 7* | IPO8 | importin 8 | nuclear protein import | s20635 | CAUUCAACAUUCACGAAAAtt | UUUUCGUGAAUGUUGAAUGga | Tubular |
| 8 | SRSF1 | splicing factor,  arginine/serine-rich 1 | Ensures the accuracy of splicing and regulating alternative splicing. | s12727 | GCAUCUACGUGGGUAACUUtt | AAGUUACCCACGUAGAUGCgg | Fragmented^✝^ |
| 9 | XWNeg9 |  | negative control | s444246 | UACGACCGGUCUAUCGUAGtt | CUACGAUAGACCGGUCGUAtt |  |
| 10 | NT5C | 5',3'-nucleotidase, cytosolic | Catalyzes the dephosphorylation of the 5' deoxyribonucleotides (dNTP) and 2'(3')-dNTP and ribonucleotides | s195191 | GCUUUUUCCUGGACCUGGAtt | UCCAGGUCCAGGAAAAAGCcc | Condensed^✝^ |
| 11* | ACTR3 | ARP3 actin-related protein 3 homolog | ATP-binding component of the Arp2/3 complex | s19642 | GGACGAGAUAUAACAUAUUtt | AAUAUGUUAUAUCUCGUCCtg | Condensed |

| 12 | PTBP1 | polypyrimidine tract  binding protein 1 | Involved in mRNA metabolism and transport | s11436 | GCAUCACGCUCUCGAAGCAtt | UGCUUCGAGAGCGUGAUGCgg | Condensed^✝^ |
| --- | --- | --- | --- | --- | --- | --- | --- |
| 13* | DNM1 | dynamin 1 | Dynamin subfamily of GTPbinding proteins, involved in clathrin-mediated endocytosis and other vesicular trafficking processes | s144 | GCAGUUCGCCGUAGACUUUtt | AAAGUCUACGGCGAACUGCtg | Fragmented |
| 14 | FAM177B | family with sequence  similarity 177,  member B |  | s53382 | AGACUACUCCUAAAAGGAUtt | AUCCUUUUAGGAGUAGUCUtt | Tubular^✝^ |
| 15 | PTBP1 | polypyrimidine tract  binding protein 1 | Involved in mRNA metabolism and transport | s11436 | GCAUCACGCUCUCGAAGCAtt | UGCUUCGAGAGCGUGAUGCgg | Condensed^✝^ |
| 16 | ARHGAP44 | Rho GTPase  activating protein 44 | GTPase-activating protein (GAP) that stimulates the GTPase activity of Rho-type GTPases. | s19218 | AGAACCUCUUAUGACCUUUtt | AAAGGUCAUAAGAGGUUCUgg | Tubular^✝^ |
| 17 | XWNeg9 |  | Negative control | s444246 | UACGACCGGUCUAUCGUAGtt | CUACGAUAGACCGGUCGUAtt |  |
| 18* | ACTR3 | ARP3 actin-related protein 3 homolog | ATP-binding component of the Arp2/3 complex | s19642 | GGACGAGAUAUAACAUAUUtt | AAUAUGUUAUAUCUCGUCCtg | Condensed |
| 19 | SRSF1 | splicing factor,  arginine/serine-rich 1 | Ensures the accuracy of splicing and regulating alternative splicing | s12727 | GCAUCUACGUGGGUAACUUtt | AAGUUACCCACGUAGAUGCgg | Fragmented^✝^ |
| 20 | C1S | complement  component 1, s subcomponent | Encodes a serine protease, which is a major constituent of the human complement  subcomponent C1 | s2157 | CCAAGUCCCAUACAACAAAtt | UUUGUUGUAUGGGACUUGGaa | Tubular^✝^ |
| 21* | IPO8 | importin 8 | nuclear protein import | s20635 | CAUUCAACAUUCACGAAAAtt | UUUUCGUGAAUGUUGAAUGga | Tubular |
| 22 | WDR75 | WD repeat domain 75 | Ribosome biogenesis factor | s38530 | CAGCUAGCAAAGAUGGUUAtt | UAACCAUCUUUGCUAGCUGta | Condensed^✝^ |
| 23 | NT5C | 5',3'-nucleotidase, cytosolic | Catalyzes the dephosphorylation of the 5' deoxyribonucleotides (dNTP) and 2'(3')-dNTP and ribonucleotides | s195191 | GCUUUUUCCUGGACCUGGAtt | UCCAGGUCCAGGAAAAAGCcc | Condensed^✝^ |
| 24 | FAM177B | family with sequence  similarity 177,  member B |  | s53382 | AGACUACUCCUAAAAGGAUtt | AUCCUUUUAGGAGUAGUCUtt | Tubular^✝^ |
| 25* | COPB1 | coatomer protein  complex, subunit beta 1 | Coatomer complex required for budding from Golgi membranes and essential for the retrograde Golgi-to-ER transport of dilysine tagged proteins | s3371 | GGUCUGUCAUGCUAAUCCAtt | UGGAUUAGCAUGACAGACCtt | Diffuse |
| 26 | ARHGAP44 | Rho GTPase  activating protein 44 | GTPase-activating protein (GAP) that stimulates the GTPase activity of Rho-type GTPases. | s19218 | AGAACCUCUUAUGACCUUUtt | AAAGGUCAUAAGAGGUUCUgg | Tubular^✝^ |
| 27 | XWNeg9 |  | negative control |  |  |  |  |
| 28 | GPT | glutamic-pyruvate transaminase (alanine aminotransferase) | Plays a key role in the intermediary metabolism of glucose and amino acids. | s6103 | CAGUUCCACUCAUUCAAGAtt | UCUUGAAUGAGUGGAACUGcg | Condensed^✝^ |
| 29 | AURKB | Aurora B Kinase | positive transfection control | s17612 | UCGUCAAGGUGGACCUAAAtt | UUUAGGUCCACCUUGACGAtg | Multinucleated |
| 30 | GPT | glutamic-pyruvate transaminase (alanine aminotransferase) | Plays a key role in the intermediary metabolism of glucose and amino acids. | s6103 | CAGUUCCACUCAUUCAAGAtt | UCUUGAAUGAGUGGAACUGcg | Condensed^✝^ |
| 31 | DENND4C | DENN/MADD domain containing 4C | Guanine nucleotide exchange factor (GEF) activating RAB10. Promotes the exchange of GDP to GTP. | s31214 | GUUUGGACCUUCCUAGUAAtt | UUACUAGGAAGGUCCAAACgt | Fragmented^✝^ |
| 32 | KIF11 | kinesin family protein 11 | positive transfection control | s7903 | GACUGAUCUUCUAAGUUCAtt | UGAACUUAGAAGAUCAGUCtt | No cells |

*Genes with highlighted examples in Figure 4

^✝^ Genes represented a variety of listed phenotypes giving a trend for this phenotype
