# Supplementary material for: CLEMSite, a software for automated phenotypic screens using light microscopy and FIB-SEM
Source: J Cell Biol. 2022 Dec 23;222(3):e202209127. doi: 10.1083/jcb.202209127 (PMC9802685; doi:10.1083/jcb.202209127)
Supplement: Table S3 — shows number of volumes targeted and acquired per dataset. [file JCB_202209127_TableS3.docx]

***Supplementary Table 3:*** **Number of volumes targeted and acquired per dataset.** The number of cells targeted in EM and successfully acquired for the three solid-phase transfection experiments are shown. The average acquisition time per cell was 35 minutes for preparation (cell location and coincidence point: 5 minutes, trench milling: 20 minutes, autofocus: 10 minutes), and around 4.5 hours per acquisition of 100 slices across the cell. The difference between acquired cells in EM and targeted cells in LM is due to either proximity between cells (once one cell is acquired, the surrounding area in a 250-300 micrometers radius becomes unusable for automatic targeting), losses due to sample preparation (e.g. crack in the resin block), or left out because of lack of time, since each session was performed usually during 8 days period.

We counted if, for any imperative reason, the current acquisition had to be stopped (“Stops during automation of run”). This event occurred for two reasons. First, it was necessary to apply heating of the gallium source to continue producing a coherent beam. In the microscope we used, the ion beam flux was stable for approximately 3 consecutive days of continuous working. The need for gallium source heating happened twice every session since each session duration was 8 days. Second, some stops were due to technical problems with the hardware, and independent of the software we developed. For example, in experiment I, a defective ESB detector forced us to stop the run and wait for technical service to repair it. It is important to remark that the software was a prototype in continuous improvement between experiments, so different versions were used in each session, causing different types of failure.

*Note: The number in parenthesis after the experiment is used to reference the experiment.*

|  | ***Targeted cells from LM*** | ***EM datasets acquired*** | ***Stops during automation of run*** |
| --- | --- | --- | --- |
| *Experiment I (****1304)*** | 29 | 21 | 3 |
| *Experiment II (****1910****)* | 34 | 20 | 1 |
| *Experiment III (****2011)*** | 44 | 36 | 4 |
