# Supplementary material for: CLEMSite, a software for automated phenotypic screens using light microscopy and FIB-SEM
Source: J Cell Biol. 2022 Dec 23;222(3):e202209127. doi: 10.1083/jcb.202209127 (PMC9802685; doi:10.1083/jcb.202209127)
Supplement: Table S4 — shows number of failures and their cause. In addition, from the acquired volumes in the second column, the following failures were a cause of rejection of the targeted cell. [file JCB_202209127_TableS4.docx]

***Supplementary Table 4*: Number of failures and their cause.** In addition, from the acquired volumes in the second column, the following failures were a cause of rejection of the targeted cell:

|  | ***Experiment I (1304)*** | ***Experiment II (1910)*** | ***Experiment III (2011)*** |
| --- | --- | --- | --- |
| *ROI incorrectly targeted (shifted)* | 2 | 3 | 4 |
| *ROI fully missing* | 0 | 1 | 1 |
| *Wrong LM selection: Mitotic, multinucleated, or absent* | 4 | 1 | 4 |
| *Out of focus* | 2 | 7 | 0 |
| *Scratches in the sample* | 0 | 0 | 2 |
| **TOTAL** | 8/21 | 12/20 | 11/36 |
